# Impact of a history of cardiovascular disease and physical activity habits on the incidence of functional disability

**DOI:** 10.1038/s41598-023-47913-z

**Published:** 2023-11-27

**Authors:** Remi Kodera, Kazuya Fujihara, Tetsuya Koyama, Haruka Shiozaki, Yurie Mutsuma, Noriko Yagyuda, Mariko Hatta, Kahori Tsuruoka, Yasunada Takeda, Atsushi Araki, Hirohito Sone

**Affiliations:** 1 Department of Internal Medicine, Niigata University Faculty of Medicine, 1-757, Asahimachi, Niigata, Niigata Japan; 2 Department of Diabetes, Metabolism, and Endocrinology, Tokyo Metropolitan Institute for Geriatrics and Gerontology, 35-2, Sakaecho, Itabashi-ku, Tokyo, Japan

**Keywords:** Cardiology, Diseases, Health care, Medical research, Neurology

## Abstract

We examined the impact of a history of coronary artery disease (CAD) or cerebrovascular disease (CVD) and physical activity habits on functional disability among community-dwelling Japanese adults. This population-based retrospective cohort study included 10,661 people aged 39–98 years in Japan (5054, men). Median follow-up was 3.7 years. During the study period, 209 functional disabilities occurred in the overall study population. In multivariable analysis, a history of CVD (hazard ratio [HR] 1.57 [95% CI: 1.00–2.45]) and no physical activity habit (HR 1.74 [1.27–2.39]) presented increased risks for functional disability. HRs for functional disability among patients with a CVD history with and without a physical activity habit were 1.68 (0.75–3.74) and 2.65 (1.49–4.71), respectively, compared with individuals without a history of CVD with a physical activity habit. Similar results were observed for CAD. We found no significant difference in the incidence of functional disability between the group with a history of CAD or CVD and physical activity habits and the group with no history of CAD or CVD and without physical activity habits. Physical activity habits had a favorable influence on avoiding functional disability regardless of a history of CAD or CVD. Future prospective studies are needed to clarify these associations.

## Introduction

Life expectancy and a healthy life expectancy have increased by 6.78 years and 5.66 years, respectively, between 1995 and 2017 according to a study that included data from 195 countries^1^. Similarly, both life expectancy and healthy life expectancy for Japanese have been extended in the past 15 years^2,3^.

A recent report from the World Health Organization noted that adding more years to life can be a mixed blessing if it is not accompanied by adding more life to years^4^. In other words, increasing a healthy life expectancy, that is, life without functional disability, is considered more important than increasing the average life expectancy. There is about a 10-year difference between life expectancy and a healthy life expectancy^1–3^. Shortening this gap is relevant and urgent not only to expand healthy life expectancy but also to reduce the enormous socioeconomic impact of poor health in the final years^5–7^. Since declining health due to functional disability makes independent living in the community more difficult^8–10^, clarifying the risk factors for functional disability that shorten healthy life expectancy is important^5,8,11,12^.

Cardiovascular disease is one of the top causes of functional disability and influences health care systems and economies worldwide^13–15^. In addition, cardiovascular disease accelerates the risk of functional disability through the recurrence of cardiovascular events^16–19^. Therefore, prevention of functional disability in individuals with a history of cardiovascular disease is essential in clinical practice^20^.

Habitual physical activity can prevent functional disability and reduce mortality^21–23^. For example, physical activity was linked to lower mortality in subsequent years regardless of prior cardiovascular disease and had a greater impact on reduced mortality in secondary prevention than in primary prevention^6^. Although some reports investigated risk factors for the incidence of functional disability in secondary prevention^5,16,24–26^, little is known about the combination of a history of cardiovascular disease and physical activity habits on the incidence of functional disability. Previous studies also showed that physical activity could reduce the risk of functional disability in those with coronary artery disease (CAD) or cerebrovascular disease (CVD)^24–26^ analyzed separately, but they have not analyzed the effect of both of these conditions in the same cohort.

Therefore, we investigated the impact of a history of cardiovascular disease, physical activity habits and their combinations on functional disability among community-dwelling Japanese adults.

## Methods

### Study participants

In Japan, care is provided by only one type of universal health insurance. For the present study, we recruited all people who obtained the universal health insurance in Sanjo City in Niigata prefecture, but included only those who underwent a blood test. Participants in this retrospective cohort study were 11,469 adults (aged 39–98 years) who had been enrolled between 1 October 2012 and 31 March 2015 and who had been followed for at least 2 years until 31 March 2017. Data on 10,661 people without functional disability at baseline and with health examination data including blood test results were analyzed. Participants were advised not to eat for at least 10 h prior to the blood test. They were also instructed not to consume alcohol or engage in strenuous exercise the day before the blood test. Blood was analyzed with standard methods in clinical laboratories under the nationally certified laboratory management system. If blood was drawn from those who had not fasted, plasma glucose data were treated as random. Details of the study were described elsewhere^7^.

### Definition of non-communicable diseases and physical activity habits

Participants were classified as having diabetes, hypertension and/or dyslipidemia based on HbA1c, systolic blood pressure (SBP), diastolic blood pressure (DBP), serum lipid levels and claims database data. Presence of a physical activity habit was defined according to either a ‘yes’ or ‘no’ response to the following question: “Do you perform exercise of moderate intensity at least twice a week for 30 min over a period of 1 year?”. History of CAD or CVD was defined according to either a “yes” or “no” response to the following question: “Were you diagnosed or treated for stroke (cerebral hemorrhage, cerebral infarction) or heart disease (angina, myocardial infarction) by doctors?”. The presence of CAD or CVD was also determined according to claims using International Classification of Diseases 10th revision (ICD-10) codes for CAD or CVD, medications and/or medical procedures^27^. Diabetes was defined as HbA1c ≥ 6.5% and/or the current use of antihyperglycemic agents. Hypertension was defined as SBP ≥ 140 mm Hg and/or DBP ≥ 90 mm Hg or the current use of antihypertensive agents. Non-HDL cholesterol (non-HDLC) was calculated based on the difference between total cholesterol (TC) and HDL-C, and dyslipidemia was defined as a non-HDLC level of ≥ 4.4 mmol/L (170 mg/dL) or the current use of antihyperlipidemic agents.

### Definition of functional disability

We defined functional disability according to requirements for n**e**w long-term care insurance (LTCI) certification. In Japan, a compulsory social LTCI system was introduced in 2000^28,29^. This system is the only one type of universal health insurance in Japan. All persons over the age of 40 years are automatically required to obtain the insurance; this system offers services to people aged ≥ 65 years who are certified as having functional disability and to those aged 40–64 years with specific medical conditions, such as cerebrovascular disease, diabetic complication, or requiring support in the activities of daily living. The selection process for classifying dependent adults first involves a questionnaire that evaluates the person’s current mental and physical conditions, which is analyzed using a computerized algorithm^28–30^. A long-time care approval board makes a final decision on the care that would be provided based on the algorithm-aided analysis of the questionnaire, a doctors’ recommendation and a home visit report. LTCI has seven care levels: Support Required 1–2 and Long-term care levels 1–5. The lowest level of need for care is Support Required 1 while the highest level is Long-term care level 5. Levels of disabilities designated by the LTCI program are shown in Supplementary Table 1. We defined participants as being without functional disability if they had not applied for care through the insurance at baseline.

### Statistical analysis

Continuous variables were expressed as means ± SD. Categorical variables were expressed as numerals and percentages. For comparisons between groups of cases and non-cases, χ^2^ tests were used for the categorical variables. Unpaired Student’s t-test was used for the continuous variables. Cox regression model identified variables related to the incidence of functional disability. Covariates included traditional risk factors for functional disability in each model: age, sex, body mass index (BMI) category, diabetes, hypertension, dyslipidemia, physical activity habits, smoking status, history of CAD and history of CVD. The start date was the earliest year for which health examination data existed, and the end date was the last year during which we could follow up. We also defined the date in which the level of long-term care insurance was determined as the “event” and the last date in which we could not track as the “censor”. Unadjusted overall time to development of functional disability was derived by a cumulative incidence rate curve and log-rank tests. Analyses were performed using SPSS V.19.0. Statistical significance was considered for p values < 0.05.

### Ethics statement

This study was conducted according to the guidelines of the Declaration of Helsinki on research involving human subjects. This study was approved by the Ethics Committee of the Niigata University (2015-2355) and the requirement for informed consent was waived. Although we could not obtain signed informed consent for the use of data from all participants, an announcement describing the study was made through the internet, including the information that participants could opt out regarding use of their data.

## Results

The median observation period was 3.67 years. At baseline 688 adults had a history of CAD and 453 adults had a history of CVD. New functional disabilities developed in 209 participants during the study period.

**Figure 1:**
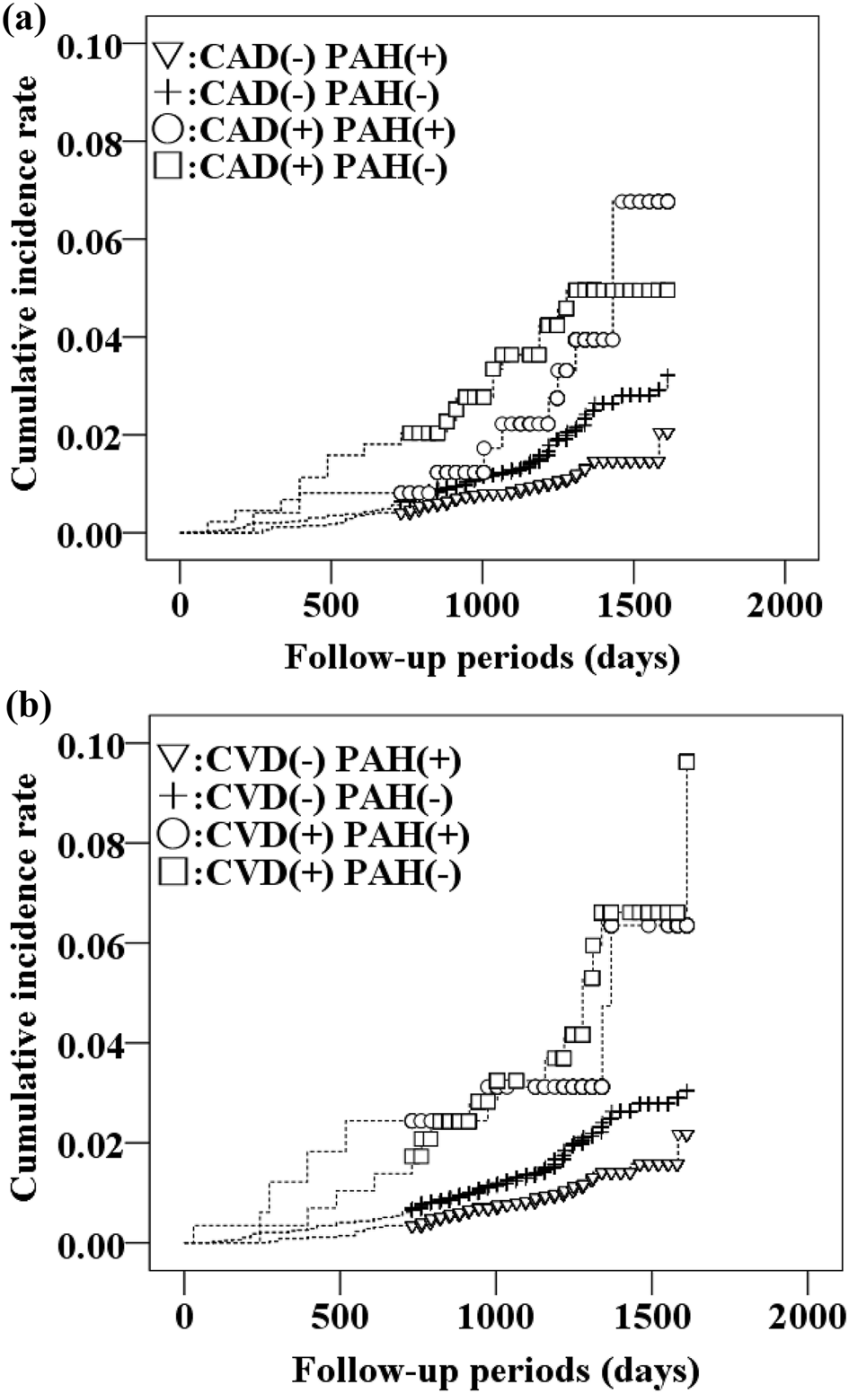
Cumulative incidence rates of functional disability. (**a**) Combination of CAD and PAH, (**b**) combination of CVD and PAH. CAD, coronary artery disease; CVD, cerebrovascular disease; PAH, physical activity habit.

Baseline characteristics of those with and without CAD/CVD are summarized in Table 1. Participants with a history of CAD/CVD were significantly older and included a higher percentage of men, had higher BMI and HbA1c values, and greater rates of diabetes, hypertension and dyslipidemia compared with those without a history of CAD/CVD. The percentages of those with a physical activity habit were similar between the two groups. Values for non-HDL and TC and percentage of current smoking were lower in individuals with than without a history of CAD/CVD. Supplementary Table 2 shows the baseline characteristics of those with and without physical activity habits. Some statistical differences disappeared due to the small number of events, but the trends did not change. Multivariable analysis showed that age in 5-year increments (HR 2.38 [95% confidence interval (CI) 2.16–2.61]), diabetes (HR 1.57 [1.06–2.33]), no physical activity habit (HR 1.74 [1.27–2.39]), history of CVD (HR 1.57 [1.00–2.45]), and low BMI category (> 18.5 kg/m^2^) (HR 1.75 [1.13–2.70]) presented increased risks for functional disability (Table 2). The HRs for BMI < 18.5 kg/m^2^ and a history of CVD were the same as for a no physical activity habit. Figure 1 shows the cumulative incidence rates for functional disability according to the combination of the presence or absence of a history of CAD/CVD and a physical activity habit. The risks for functional disability increased in individuals with a history of CAD/CVD who did not have a physical activity habit, whereas those relationships were attenuated in individuals with a history of CAD/CVD who had a physical activity habit (Table 3). HRs for functional disability among patients with a CVD history with and without a physical activity habit were 1.68 (0.75–3.74) and 2.65 (1.49–4.71), respectively, compared with individuals without a history of CVD but with a physical activity habit. Similarly, HRs for functional disability among patients with a CAD history with and without a physical activity habit were 1.34 (0.66–2.84) and 2.05 (1.19–3.53), respectively, compared with individuals without history of CAD but with a physical activity habit. For results stratified by sex in Supplementary Tables 3 and 4, some statistical differences disappeared due to the small number of events, but the trends did not change.

**Table 1:** Characteristics of study participants according to the presence or absence of a history of CAD and CVD.

	CAD			CVD		
	(−)	(+)	p value	(−)	(+)	p value
	n = 9973	n = 688		n = 10,208	n = 453	
Male sex (%)	4605 (46)	419 (61)	< 0.001	4748 (47)	276 (61)	< 0.001
Age (years)	66 ± 10	71 ± 8	< 0.001	66 ± 10	71 ± 8	< 0.001
BMI (kg/m^2^)	22.7 ± 3.1	23.3 ± 3.2	< 0.001	22.7 ± 3.1	23.4 ± 3.2	< 0.001
< 18.5	770 (8)	32 (5)		778 (8)	24 (5)	
18.5–25.0	7149 (72)	475 (69)		7326 (72)	298 (66)	
≥ 25.0	2054 (21)	181 (26)		2104 (21)	131 (29)	
Systolic blood pressure (mm Hg)	127 ± 17	128 ± 17	0.478	127 ± 17	129 ± 17	0.105
Diastolic blood pressure (mm Hg)	75 ± 11	73 ± 12	< 0.001	74 ± 11	73 ± 12	0.431
HbA1c (%)	5.7 ± 0.6	5.8 ± 0.6	< 0.001	5.7 ± 0.6	5.8 ± 0.6	< 0.001
Total cholesterol (mmol/L)	5.2 ± 0.8	4.9 ± 0.8	< 0.001	5.2 ± 0.8	4.9 ± 0.8	< 0.001
HDL cholesterol (mmol/L)	1.5 ± 0.4	1.5 ± 0.4	< 0.001	1.5 ± 0.4	1.5 ± 0.4	0.028
Non-HDL cholesterol (mmol/L)	3.7 ± 0.8	3.4 ± 0.8	< 0.001	3.7 ± 0.8	3.4 ± 0.8	< 0.001
Diabetes (%)	852 (9)	93 (14)	< 0.001	863 (9)	82 (18)	< 0.001
Hypertension (%)	4228 (42)	471 (69)	< 0.001	4339 (43)	300 (66)	< 0.001
Dyslipidemia (%)	3534 (35)	283 (41)	0.003	3625 (36)	192 (42)	0.003
Walking speed (%)	4780 (48)	257 (37)	< 0.001	4876 (48)	161 (36)	< 0.001
Physical activity habit (%)	3439 (35)	246 (36)	0.497	3521 (35)	164 (36)	0.454
Daily activity (%)	5613 (56)	344 (50)	0.001	5714 (56)	243 (54)	0.328
Disability (%),	181 (2)	28 (4)	< 0.001	186 (2)	23 (5)	< 0.001
Current smoking (%)	1368 (14)	55 (8)	< 0.001	1380 (14)	43 (10)	0.014

Data are presented as numbers, means ± SDs or percentages.

CAD, coronary artery disease; CVD, cerebrovascular disease; BMI, body mass index; HbA1c, hemoglobin A1c; HDL, high-density lipoprotein; SD, standard deviation.

**Table 2:** Cox regression analysis of variables for the incidence of functional disability.

	HR (95% CI)	*p* value
Age, 5-y increase	2.38 (2.16–2.61)	< 0.001
Male sex	1.26 (0.94–1.68)	0.129
BMI (kg/m^2^)		
< 18.5	1.75 (1.13–2.70)	0.012
18.5–24.9	ref	
≥ 25.0	0.93 (0.65–1.33)	0.671
Diabetes	1.57 (1.06–2.33)	0.025
Hypertension	1.30 (0.97–1.74)	0.077
Dyslipidemia	0.83 (0.61–1.13)	0.228
History of CAD	1.17 (0.78–1.76)	0.460
History of CVD	1.57 (1.00–2.45)	0.047
No physical activity habit	1.74 (1.27–2.39)	0.001
Current smoking	1.19 (0.71–2.01)	0.508

BMI, body mass index; CAD, coronary artery disease; CVD, cerebrovascular disease; HR, hazard ratio.

**Table 3:** Hazard ratios with 95% confidence intervals for functional disability analyzed by Cox models according to risk of combinations of CAD and physical activity habit (A) and CVD and physical activity habit (B).

(A)			
	Cases/total	HR (95% CI)	*p* value
CAD (−) physical activity habits (+)	43/3439	1.00 (ref)	
CAD (−) physical activity habits (−)	138/6534	1.78 (1.26 to 2.51)	< 0.001
CAD (+) physical activity habits (+)	9/246	1.34 (0.67 to 2.84)	0.391
CAD (+) physical activity habits (−)	19/442	2.05 (1.19 to 3.53)	0.010

(B)			
	Cases/total	HR (95% CI)	*p* value
CVD (−) physical activity habits ( +)	45/3521	1.00 (ref)	
CVD (−) physical activity habits (−)	141/6687	1.76 (1.26 to 2.47)	< 0.001
CVD (+) physical activity habits (+)	7/164	1.68 (0.75 to 3.74)	0.209
CVD (+) physical activity habits (−)	16/289	2.65 (1.49 to 4.71)	< 0.001

Adjusted for age, sex, body mass index, diabetes, hypertension, dyslipidemia, current smoking.

CAD, coronary artery disease; CVD, cerebrovascular disease; HR, hazard ratio.

## Discussion

Although previous studies reported that physical activity could reduce the risk of functional disability for CVD and CAD, respectively, we analyzed these two conditions together. We showed that even for patients with CVD/CAD, physical activity may reduce the risk of functional disability to the same level as in those without CVD/CAD. In contrast, no physical activity habit increased the incidence of functional disability regardless of the presence or absence of a history of cardiovascular disease.

A history of CAD did not significantly increase the risk of functional disability, whereas a history of CVD was significantly associated with an increased risk of functional disability. Previous studies showed an association between CAD/CVD and functional disability^14,31–33^. Ford et al. reported that stroke, visual impairment, heart disease and dementia accounted for more than half of causes of functional disability^32^. According to the Ministry of Health, Labour and Welfare's National Survey of Basic Living Conditions in 2016, the main causes of the need for nursing care for those aged ≥ 65 were dementia at 18.7%, followed by CVD at 15.1%, weakness due to aging at 13.8%, fractures and falls at 12.5%, joint diseases at 10.7% and cardiac disease at 4.7%^34^. Since CVD is more likely to cause paralysis and dysphagia than CAD, the association of CVD with functional disability can be considered to be more likely than that with CAD. Furthermore, in a large population-based study, the trajectory of increasing disability became significantly steeper after stroke but not after myocardial infarction^35^. In addition, functional disability after stroke could be progressive and long-lasting. Vascular risk factors can cause not only recurrent strokes but also subclinical brain damage that manifests as infarction and may lead to functional disability^36^. Stroke also causes an increased systemic inflammatory profile, resulting in damaged brain structure and function^37^. Moreover, reduced metabolic fitness due to static functional disability is likely to occur more frequently after stroke than after myocardial infarction^38^. Taken together, CVD is more likely to cause functional disability compared with CAD.

The main reason that a history of CAD was not shown to be a significant risk for functional disability may be due to differences in the definition of CAD between our study and previous studies^25,39,40^. Our definition of CAD did not include heart failure, whereas heart failure accounted for 27.6% of coronary heart disease in a previous study^11^. Moreover, the mean age of our study participants was lower than in previous studies^5,41^. Aging has a significant impact on the onset and progression of CAD^13^. In addition, the impact of a functional decline in those with CAD is slower than with CVD^13^. These reasons might account for the findings of a low impact of CAD on functional disability.

The risk for functional disability was significantly reduced in groups having physical activity habits. Cunningham et al. reported a 22% reduction in mortality from all causes in the group having low level physical activity (1–499 MET-min/week) compared with the inactive group (0 MET-min/week)^42^. In addition, in another study, a group with moderate physical activity habits (3–5 days/week and 30 min per day) had a 30% lower risk of functional disability compared with the inactive group^43–45^. Taken together, physical activity habits have been shown to reduce conditions due to non-communicable diseases such as arteriosclerosis and frailty, leading to prevention of cardiovascular diseases or to maintenance of muscle strength^46^^.^

The percentage of people who habitually engaged in physical activity was low regardless of whether they had functional disabilities suggesting the necessity of promoting physical activity habits. Kumar et al.^47^ showed that recommendations by doctors had a significant impact on the participation in and continuation of cardiac rehabilitation. Kraus et al.^24^ also reported that the risk of mortality was reduced in persons engaging in one to two rehabilitation sessions per week compared with inactive study participants. Therefore, physicians should question their patients regarding exercise habits. Unfortunately, we could not distinguish between participants who were unable to exercise sufficiently due to cardiovascular disease and participants with a history of cardiovascular disease who started regular exercise as recommended by a physician. Thus, future study is needed to clarify these points.

The HR for functional disability among patients with a history of CAD and physical activity habits was 1.68 (0.75–3.74) compared to those with no history of CAD but with physical activity habits, which was not significantly different (p = 0.209). Similarly, the HR among patients with a history of CVD and a physical activity habit was 1.34 (0.67–2.84) compared to those with no history of CVD but with physical activity habits (p = 0.391). Also, the HR for functional disability among patients with no history of CAD and without a physical activity habit was 1.78 (1.26–2.51) compared to those with no history of CAD but with physical activity habits (p < 0.001). Similarly, the HR among patients with no history of CVD and without a physical activity habit was 1.76 (1.26–2.47) compared to those with no history of CVD but with physical activity habits (p < 0.001). We found no significant difference in the incidence of functional disability between the group with a history of CAD/CVD and physical activity habits and the group with no history of CAD/CVD and without physical activity habits. In other words, physical activity habits reduced the incidence of functional disability in patients with a history of CAD/CVD. Conversely, the risk of functional disability increased in those who had no physical activity habits even in people without a history of CAD/CVD. It was previously reported that low physical activity increased the risk of impairment of activities in daily living among the elderly^48^. A cardiopulmonary training intervention in patients with CVD reduced the risk of functional disability^49^. Our findings showed similar trends in people with a history of CAD or a history of CVD. Physical activity habits may prevent the onset of functional disability through the reduction of frailty, fractures and dementia, which are the most common risk factors for functional disability^42,49–53^. In addition, cardiovascular disease increased the risk of functional disability due to recurrence^3^. Thus, physical activity habits may contribute to the maintenance and improvement of muscle strength and cardiopulmonary function, leading to suppression of the recurrence of cardiovascular disease.

This study’s strengths were its large sample size and accurate definitions of CAD/CVD history and functional disability using data from health examinations and a claims and administrative database. These sources allowed us to precisely identify almost all patients with those conditions during follow-up. This study has some limitations. First, the design was retrospective, and only data on participants who underwent physical examinations with blood tests were analyzed. Since medical checkups were required for the construction of this database, we could not compare characteristics between those who underwent medical checkups and those who did not. Thus, we could not avoid selection bias. Second, physical activity habits were measured using a simple one-item questionnaire rather than more precise measurements such as with an acceleration sensor, which may have introduced measurement error. The following question was asked during the Japanese health examination: “Do you engage in light, sweaty exercise for at least 30 min at least 2 days a week?” To acquire information on exercise with an intensity of at least 3 METs (generally about 4 METs), “sweaty” was included in the question. This question is commonly used in Japan, but it is not used as an objective method to evaluate exercise worldwide. We also did not have detailed data on the means of physical activity such as aerobic, balance, and/or resistance training and their combinations. Moreover, there was no way to identify those participants whose physical activity had either improved or deteriorated during follow-up. Thus, physical activity might be over- or underestimated. Third, during the follow-up period, 99 people died. Of these, 36 people were using long-term care. Although this did not significantly affect the results of the analysis, it could be considered a bias. Fourth, we could not assess in detail why the participants needed LTCI services. Thus, further detailed investigations are needed that include data on the main causes for needing LTCI services, such as those on social relationships and socioeconomic status such as employment status and/or income. Finally, we did not have data on anthropometric measurements, dietary patterns, mental health, socioeconomic status and social frailty such as living alone, going out infrequently and having friends. Thus, future studies are necessary to confirm our findings considering these important factors. In conclusion, there are confounding factors in this study that must be resolved. In addition, an intervention study must be conducted to determine if functional disability can improve and under what conditions.

## Conclusion

In summary, a history of CVD and lack of a physical activity habit were independently associated with functional disability among Japanese adults. Physical activity habits had a favorable influence on avoiding functional disability regardless of a history of CAD or CVD. Clinicians should encourage their patients to exercise in clinical settings regardless of a history of cardiovascular disease. This is important not only for the prevention of lifestyle-related diseases, but also for the avoidance of long-term care. As a result, healthy life expectancy would increase. Future interventional studies focusing on physical activity in these groups are needed to apply our findings to clinical practice.

### Supplementary Information

Supplementary Tables.

## Data Availability

The data that support the findings of this study are available on request from the corresponding author. The data are not publicly available due to privacy reasons.
