# Supplementary material for: Impact of a history of cardiovascular disease and physical activity habits on the incidence of functional disability
Source: Sci Rep. 2023 Nov 27;13:20793. doi: 10.1038/s41598-023-47913-z (PMC10682401; doi:10.1038/s41598-023-47913-z)
Supplement: Supplementary file 1 — Supplementary Tables. [file 41598_2023_47913_MOESM1_ESM.docx]

**Supplementary Table 1. Definitions of levels of services required by long-term care insurance in Japan**

|  | ADL Conditions |
| --- | --- |
| Support  Required 1 | Long-term care is needed for some aspects of daily living such as cooking, shopping and taking oral medicine. Proper care such as provided by the LTCI can improve or maintain ADL. |
| Support Required 2 | The person’s ability to handle task-based activities in daily life is slightly lower than that of individuals in the Support Required 1 category. |
| Long-term Care Level 1 | Unstable in rising and gait; partial support needed in toileting, bathing, etc. |
| Long-term Care Level 2 | Difficulty in rising and gait; partial or complete support needed in toileting, bathing, etc. |
| Long-term Care Level 3 | Impossible to rise and cannot walk. Complete support needed in toileting, bathing, dressing, and all other basic ADL. |
| Long-term Care Level 4 | Severe decline in ADL capacity; complete support needed in toileting, bathing, dressing, and all other basic ADL. |
| Long-term Care Level 5 | Complete support needed in all ADL; difficulty with communication. |

Abbreviations: ADL**,** activities of daily living; LTCI, long-term care insurance

**Supplementary Table 2. Characteristics of study participants according to the presence or absence of a physical activity habit**

|  | PAH |  |  |
| --- | --- | --- | --- |
|  | (+) | (-) | P value |
|  | n=3685 | n=6976 |  |
| Male sex (%) | 1869(51) | 3155(45) | <0.001 |
| Age (years) | 67±8 | 65±10 | <0.001 |
| BMI (kg/m²) | 22.6±3.0 | 22.7±3.2 | 0.017 |
| <18.5 | 255(7) | 547(8) |  |
| 18.5-25.0 | 2729(74) | 4895(70) |  |
| ≥25.0 | 701(19) | 1534(22) |  |
| Systolic blood pressure  (mm Hg) | 128±17 | 127±17 | 0.238 |
| Diastolic blood pressure  (mm Hg) | 75±11 | 73±12 | 0.020 |
| HbA1c (%) | 5.7±0.6 | 5.7±0.6 | <0.001 |
| Total cholesterol(mmol/L) | 5.2±0.8 | 5.2±0.9 | 0.822 |
| HDL cholesterol (mmol/L) | 1.6±0.4 | 1.5±0.4 | <0.001 |
| Non-HDL cholesterol (mmol/L) | 3.6±0.8 | 3.7±0.8 | 0.091 |
| Diabetes (%) | 390(11) | 555(8) | <0.001 |
| Hypertension (%) | 1636(44) | 3063(44) | 0.629 |
| Dyslipidemia (%) | 1318(36) | 2499(36) | 0.954 |
| History of CAD (%) | 246(7) | 442(6) | 0.497 |
| History of CVD (%) | 164(5) | 289(4) | 0.454 |
| Walking speed (%) | 2328(63) | 2709(39) | <0.001 |
| Daily activity (%) | 2826(77) | 3131(45) | <0.001 |
| Disability (%) | 52(1) | 157(2) | 0.003 |
| Current smoking (%) | 414(11) | 1009(15) | <0.001 |

Data are presented as numbers, means ± SDs or percentages.

Abbreviations: PAH, physical activity habit; CAD, coronary artery disease; CVD, cerebrovascular disease; BMI, body mass index; HbA1c, hemoglobin A1c; HDL, high-density lipoprotein; SD, standard deviation

**Supplementary Table 3. Cox regression analysis of variables for the incidence of functional disability stratified by sex**

|  | Male |  | Female |  |
| --- | --- | --- | --- | --- |
|  | HR (95% CI) | *p* value | HR (95% CI) | *p* value |
| Age, 5-y increase | 2.28 (2.00-2.60) | <0.001 | 2.51 (2.18-2.89) | <0.001 |
| BMI (kg/m²) |  |  |  |  |
| <18.5 | 2.71 (1.48-4.95) | 0.001 | 1.20 (0.64-2.25) | 0.567 |
| 18.5-24.9 | ref |  | ref |  |
| ≥25.0 | 0.90 (0.56-1.45) | 0.664 | 0.95 (0.55-1.64) | 0.859 |
| Diabetes | 1.38 (0.83-2.29) | 0.220 | 1.92 (1.03-3.58) | 0.041 |
| Hypertension | 1.38 (0.93-2.06) | 0.110 | 1.18 (0.76-1.82) | 0.471 |
| Dyslipidemia | 0.94 (0.60-1.46) | 0.766 | 0.74 (0.48-1.13) | 0.165 |
| History of CAD | 1.10 (0.64-1.88) | 0.744 | 1.34 (0.71-2.53) | 0.369 |
| History of CVD | 1.60 (0.91-2.83) | 0.103 | 1.60 (0.77-3.35) | 0.047 |
| No physical activity habit | 1.76 (1.16-2.65) | 0.007 | 1.66 (1.01-2.73) | 0.046 |
| Current smoking | 1.15 (0.66-1.99) | 0.627 | 0.86 (0.12-6.22) | 0.879 |

Abbreviations: BMI, body mass index; CAD, coronary artery disease; CVD, cerebrovascular disease; HR, hazard ratio

**Supplementary Table 4. Hazard ratios with 95% confidence intervals for functional disability analyzed by Cox models according to risk of combinations of CAD and physical activity habit (A) and CVD and physical activity habit (B) stratified by sex**

**A.**

|  | Male |  |  | Female |  |  |
| --- | --- | --- | --- | --- | --- | --- |
|  | Cases/total | HR (95% CI) | *p* value | Cases/total | HR (95% CI) | *p* value |
| CAD (-)PAH(+) | 26/1710 | 1.00 (ref) |  | 17/1729 | 1.00 (ref) |  |
| CAD (-) PAH(-) | 71/2895 | 1.78 (1.14 to 2.80) | 0.012 | 67/3639 | 1.76 (1.03 to 3.01) | 0.040 |
| CAD (+)PAH(+) | 6/159 | 1.22 (0.50 to 2.99) | 0.666 | 3/87 | 1.87 (0.54 to 6.41) | 0.322 |
| CAD (+)PAH(-) | 11/260 | 1.98 (0.97 to 4.04) | 0.061 | 8/182 | 2.11 (0.90 to 4.95) | 0.086 |

**B.**

|  | Male |  |  | Female |  |  |
| --- | --- | --- | --- | --- | --- | --- |
|  | Cases/total | HR (95% CI) | *p* value | Cases/total | HR (95% CI) | *p* value |
| CVD (-)PAH(+) | 29/1767 | 1.00 (ref) |  | 16/1754 | 1.00 (ref) |  |
| CVD (-) PAH(-) | 70/2981 | 1.60 (1.04 to 2.48) | 0.034 | 71/3706 | 1.99 (1.16 to 3.44) | 0.014 |
| CVD (+)PAH(+) | 3/102 | 0.86 (0.26 to 2.86) | 0.810 | 4/62 | 5.06 (1.67 to 15.4) | 0.004 |
| CVD (+)PAH(-) | 12/174 | 3.03 (1.54 to 5.99) | 0.001 | 4/115 | 1.83 (0.60 to 5.55) | 0.286 |

Adjusted for age, sex, body mass index, diabetes, hypertension, dyslipidemia, current smoking.

Abbreviations: PAH, physical activity habit; CAD, coronary artery disease; CVD, cerebrovascular disease; HR, hazard ratio
